# Accuracy of Endotracheal Tube Cuff Pressure Adjustment by Fingertip Palpation after Training of Intensive Care Unit Nurses

**DOI:** 10.5812/ircmj.4164

**Published:** 2013-05-05

**Authors:** Aptin Maboudi, Hamidreza Abtahi, Mostafa Hosseini, Amin Tamadon, Enayat Safavi

**Affiliations:** 1 Department of Pulmonary and Critical Care Medicine, Imam Khomeini Medical Center, Tehran University of Medical Sciences, and Thoracic Diagnostic and Therapeutic Research Center, Tehran University of Medical Sciences, Tehran, IR Iran; 2 Department of Epidemiology and Biostatistics, Tehran University of Medical Sciences, Tehran, IR Iran; 3 Department of Animal Health Management, School of Veterinary Medicine, Shiraz University and Stem Cell and Transgenic Technology Research Center, Shiraz University of Medical Sciences, Shiraz, IR Iran

**Keywords:** School, Nursing, Pressure, Manometry

## Abstract

**Background:**

Untrained fingertip palpation has been shown to be unable to adjust endotracheal tube (ET) cuff pressure in most intubated patients.

**Objective:**

We determined the impact of a training course of ETT cuff adjustment by palpation in attaining a safe cuff inflation pressure.

**Patients and Methods:**

Twenty-five nurses were asked to adjust the ETT cuff pressure to 25 cmH2O by palpation on three different patients in seven sessions (S1-S7): before training, two hours and two days after first training session, and two hours, two days, one week, and eight months after second training session.

**Results:**

Before training, 41.3% of the cuff pressures were adjusted to the safe range of 21-35 cmH2O. The pressures obtained in S2-S7 were lower and less variable than S1 (P = 0.02 to < 0.0001). After the first training session, all the ETT cuff pressures in the high range (36-50 cmH2O) and 87.5% in the very high range (> 50 cmH2O) changed to the safe range.

**Conclusions:**

Using trained fingertip adjustment of the ETT cuff pressure throughout the nursing shift with protocols for the adjustment of out of range pressures at the beginning of each shift by cuff manometer could decrease the rate of ETT cuff over- and under-inflation.

## 1. Background

Proper inflation of the endotracheal tube (ETT) cuff during mechanical ventilation is of vital importance in ensuring adequate ventilation and preventing the complications of intubation. The routine measurement and adjustment of ETT cuff pressure by a cuff manometer at the start of each nursing shift and with any change in patient position or ventilation has been recommended. However, this is not routinely practiced in many ICUs. (1) Also, even after implementation of a cuff manometer protocol, ETT cuff pressure was high in at least 24% of patients (2). Fingertip palpation was considered to be imprecise for adjusting the ETT cuff pressure in most patients (3). After training, the airway care providers demonstrated more expertise in the determination of ETT cuff pressure by palpation in an in-vitro ETT model (4).

## 2. Objective

A before-and-after training prospective study was therefore conducted to determine the impact of a training course of ETT cuff pressure adjustment by fingertip palpation in attaining a safe cuff inflation pressure by ICU nurses.

## 3. Patients and Methods

**Figure 1.**
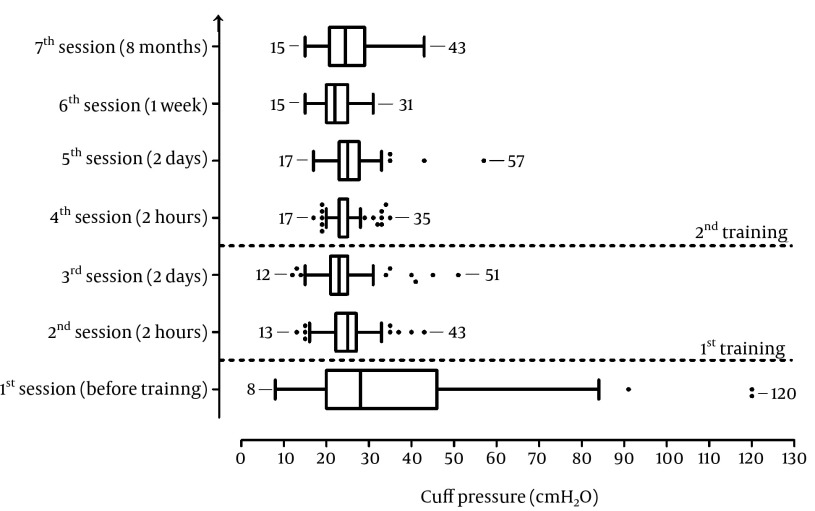
Box and whisker plot of median endotracheal cuff tube pressure (cmH2O) before and after training sessions measured by cuff manometry in the different periods of adjustment of cuff pressure by hand touching of ICU nurse training

The present study was conducted from March to December 2010 in the 16-bed ICU of Imam Khomeini Medical Center affiliated with Tehran University of Medical Sciences within seven sessions (S1 to S7, Figure 1). Twenty-five volunteer ICU nurses enrolled in this study. In each session, the nurses were asked to adjust the ETT cuff pressure to 25 cmH 2 O by feeling the pilot balloon on three random adult patients. Immediately after inflation, the cuff pressure was measured by a cuff manometer (Mallinckrodt Medical, Athlone, Ireland) and the entire out-of-range cases adjusted to 25-30 cmH 2 O. After S1 and before the first training course, the nurses were asked about the functions of the ETT cuff and the safe recommended ETT cuff pressure. Then, the ETT cuff functions and its recommended pressure range were explained. The objective of the training courses was to influence the ability of nurses to inflate the ETT cuff to a safe pressure, defined as 25 cmH 2 O, as well as, to identify excessive intra cuff pressure by palpation of the pilot balloon in previously inflated ETT cuffs. The training device was composed of a cuff manometer connected to an ETT tube (8 mm internal diameter, high volume-low pressure, SUPA Medical Devices, Tehran, Iran). The ETT was put in an opaque rubber tube with a 12 mm internal diameter used as a tracheal model. In each training course, the ETT cuff was inflated to pressures of 10, 20, 25, 30, 40, and 50 cmH 2 O. In both training courses, each of 25 nurses was permitted to become familiar with the pilot balloon feeling by fingertip palpation at different pressures until he/she was confident of being able to distinguish the pressures by his/her finger. For each of the 25 nurses, the second training course was done following S3. The institutional ethical committee approved the study. The committee waived the need for patient consent because the patients continued to receive at least their usual and customary care regardless of the study and no personal identifying information regarding the patients or ICU nurses was obtained. The data were summarized using frequencies, means ± SD, median and range percentages, or percentiles as appropriate. Four clinically relevant ETT cuff pressures, low, ≤ 20 cmH2O; safe, > 20 to ≤ 35 cmH 2 O; high, > 35 to ≤ 50 cmH 2 O and very high, > 50 cmH 2 O were defined. The means were compared by independent sample t-test. Wilcoxon signed-rank test was used to compare before/after mean pressures and pressure scores. SPSS version 18 was used and a P-value of ≤ 0.05 was considered statistically significant.

## 4. Results

The patients were intubated with 7 to 9 mm internal diameter ETTs (Supa Company). Twenty-five nurses (21 women and 4 men; mean ± SD age of 30.2 ± 4.4; 20 nurses in session 6 and 10 nurses in session 7) completed the study. Before training, seven nurses theoretically knew the safe pressure range of an ETT cuff, whereas 18 nurses with less ICU employment experience (1.9 ± 1.6 vs. 8.5 ± 7.8 years; P = 0.001) were unaware of the ETT cuff function and recommended range of ETT cuff pressure. Pressures obtained by fingertip palpation in S1 ranged from 8 to 120 cmH 2 O (36.2 ± 24.2 cmH 2 O, Table 1 and Figure. 1). Two of seven nurses who theoretically knew the safe pressure range of an ETT cuff before training, truly adjusted the ETT cuff in all three patients by fingertip palpation. The pressures obtained in S2 were lower and less variable (13 to 43 cmH 2 O) than S1 (P < 0.0001). The pressures obtained in S4 were lower and less variable (from 17 to 35 cm H 2 O) than S1 (P < 0.0001) and S3 (P = 0.02), but not than S2. In S5, S6, and S7 the pressures were lower and less variable than in S1 (P < 0.001). The cuff score changed after the training sessions from S1 to S2 (P < 0.0001) and S3 to S4 (P = 0.003). The pressure was in the high range in only one case eight months after the second training session.

## 5. Discussion

The study showed that most of our ICU nurses were unaware of the ETT cuff functions and its safe pressure range. Consistent with our findings, more than half of British ICU nurses in a survey did not know the recommended range of ETT cuff pressure ( 1 ). In another study, 38% of nurses in 11 different ICUs in South Africa believed that the only aim of the ETT cuff is to prevent extubation by maintaining the tube in the trachea, and 50% did not consider themselves sufficiently trained to manage cuff pressure monitoring ( 5 ). Before training, the majority of the nurses who knew the safe pressure range of an ETT cuff inflated the ETT cuff over or under the safe range. Regarding inaccuracy of cuff pressure adjustment by untrained palpation method, many experts advocate that ETT cuff pressure should be regularly monitored and regulated with a cuff manometer in intensive care units ( 2 , 3 ). However, using manometers to adjust ETT cuff pressure is time-consuming and, despite these recommendations, it has not been routinely used ( 6 ). Furthermore, one month after the implementation of a cuff pressure adjustment protocol using a cuff monometer during every team’s shift or after any patient manipulation, only 68% of patients had safe ETT cuff pressures (Table 2) ( 2 ). The percentage decreased to 40% three years after protocol implementation. With current recommendations on using a cuff manometer twice a day, and after each intervention on the tracheal tube, cuff pressure was in the safe range in 56% and in the high range in 29% of the time ( 7 ). In another study, after manual adjustment of ETT cuff pressure at 25 cmH 2 O, continuous recording of cuff pressure was performed for 8 hours in 101 patients ( 8 ). Fifty four percent of study patients developed cuff under inflation, 73% developed cuff over inflation, and 44% developed both. One third of patients developed underinflation or overinflation for more than 30 minutes. ETT cuff pressure decreased over time and 51.7% of cuff pressure values were out of range during 12 hour shift ( 9 ). Fingertip adjustment of the ETT cuff pressure was very convenient, and our study showed that with proper training, it could be a valuable method for applying desired ETT cuff pressure by ICU nurses. Seventy three percent of cuff pressures were within the safe range eight months after the second training session, and only one of the patients had a high ETT cuff pressure comparable to what reported with use of a cuff manometer ( 2 ). Our study showed that the palpation skill was sustainable until eight months after training. There was a tendency to increase the skill of fingertip adjustment of an in vitro ETT cuff pressure one month after training ( 4 ). In addition, this method of learning through the adjustment of the ETT cuff pressure in actual patients provided an unforgettable experience and left a strong impression on the mind of the trainees. Twenty three percent of cases had mild ETT cuff underinflation eight months after training session. Since all these cases have been immediately adjusted to the safe range, the consequences of this cuff under inflation could not be assessed in this study. However, underinflation has also been reported after the implementation of a cuff pressure adjustment protocol using a cuff monometer in 8-40% of cases. Two ETT cuff pressures less than 15 cmH 2 O in 4.7% of time have been reported, even with the use of a pneumatic device to maintain constant ETT cuff pressure. Using higher pressure target for cuff adjustment could decrease the possibility of underinflation, but this approach increases the possibility of overinflation. Using trained fingertip adjustment of the ETT cuff pressure throughout the nursing shift with protocols for adjustment of out- of range pressures at the start of each shift by cuff manometer could decrease the rate of ETT cuff over- and underinflation.

**Table 1.** Number (%) of endotracheal tube cuff pressure scores a obtained by fingertip palpation in seven sessions

Measurement Time ^a^		Safe, No. (%)	Low, No. (%)	High, No. (%)	Very High, No. (%)	Total, No.	Mean ± SD
**Day 1**	S1	31 (41.3)	21 (28.0)	7 (9.3)	16 (21.3)	75	36.2 ± 24.2
	S2	58 (80.6)	11 (15.3)	3 (4.2)	0 (0)	72	25.1 ± 5.6
**Day 3**	S3	53 (71.6)	17 (23.0)	3 (4.1)	1 (1.4)	74	23.9 ± 6.6
	S4	68 (90.7)	7 (9.3)	0 (0)	0 (0)	75	24.7 ± 3.5
**Day 5**	S5	60 (83.3)	10 (13.9)	1 (1.4)	1 (1.4)	72	25.8 ± 5.7
**Day 10**	S6	43 (71.7)	17 (28.3)	0 (0)	0 (0)	60	22.2 ± 3.4
**Month 8**	S7 ^b^	22 (73.3)	7 (23.3)	1 (3.3)	0 (0)	30	24.9 ± 6.0

^a^Cuff pressure scores: Safe, > 20 to ≤35 cmH2O; Low, ≤ 20 cmH2O; High, > 35 to ≤ 50 cmH2O; Very high, > 50 cmH2O. S1-S7 as defined in the text

^b^Only 10 nurses were available due to ICU staff turnover

**Table 2.** Percentage of endotracheal tube cuff pressure scores a obtained by Cuff manometer protocols (twice a day at fixed intervals and after each intervention on the endotracheal tube) in previous studies

Study	Measurement time	Appropriate, %	Low, %	High, %
**Duguet et al. (7)**	Not defined^ a^	56	15	29
**Jaber et al. (2)**	S1 ^b^	17	6	77
	**S2**	68	8	24
	**S3**	28	40	31
**Current study**	S1 ^c^	31 ^d^	28^ d^	41^ d^
	**S6**	64	27	9
	**S7**	57	23	20

^a^Percentage of time in each cuff pressure scores in a referral ICU with standard cuff manometer protocol. Appropriate, > 15 to ≤ 30 cmH2O; low, ≤ 15 cmH2O; high, > 30 cmH2O

^b^S1: Before protocol implementation; S2: One month after Protocol; S3: Three years after protocol. Appropriate, > 20 to ≤30 cmH2O; low, ≤ 20 cmH2O; high, > 30 cmH2O

^c^S1, S6 and S7 as defined in the text

^d^The definitions of cuff pressure scores are the same as was used by Jaber et al. (2) and are different from definitions that are used in Table 1
